# Time-varying auditory gain control in response to double-pulse stimuli in harbour porpoises is not mediated by a stapedial reflex

**DOI:** 10.1242/bio.021469

**Published:** 2017-02-15

**Authors:** Asger Emil Munch Schrøder, Kristian Beedholm, Peter Teglberg Madsen

**Affiliations:** 1 Zoophysiology, Bioscience, Aarhus University, C. F. Moellers Allé 3, Aarhus C 8000, Denmark; 2 Murdoch University Cetacean Research Unit, Murdoch University, South Street, Murdoch, Western Australia 6150, Australia

**Keywords:** Toothed whale, Echolocation, Stapedial reflex, Masking, Automatic gain control, Hearing

## Abstract

Echolocating animals reduce their output level and hearing sensitivity with decreasing echo delays, presumably to stabilize the perceived echo intensity during target approaches. In bats, this variation in hearing sensitivity is formed by a call-induced stapedial reflex that tapers off over time after the call. Here, we test the hypothesis that a similar mechanism exists in toothed whales by subjecting a trained harbour porpoise to a series of double sound pulses varying in delay and frequency, while measuring the magnitudes of the evoked auditory brainstem responses (ABRs). We find that the recovery of the ABR to the second pulse is frequency dependent, and that a stapedial reflex therefore cannot account for the reduced hearing sensitivity at short pulse delays. We propose that toothed whale auditory time-varying gain control during echolocation is not enabled by the middle ear as in bats, but rather by frequency-dependent mechanisms such as forward masking and perhaps higher-order control of efferent feedback to the outer hair cells.

## Abstract (precis)

**Summary:** Echolocating toothed whales reduce their hearing for short echo delays and unlike bats, do not use a stapedial reflex, but rather forward masking and a central mechanism to evoke the effect.

## INTRODUCTION

Echolocating bats and toothed whales navigate and hunt by emission of powerful sound pulses and subsequent auditory processing of weak returning echoes milliseconds later (Griffin, 1958; Au and Simmons, 2007; Madsen and Surlykke, 2013). Received echo levels (ELs) may, even for the same source level (SL), vary by several orders of magnitude due to differently sized targets and ranges (R) and hence transmission losses (Madsen et al. 2007). Many bats and toothed whales have been shown to compensate for the one way transmission loss by reducing the SL in a 20 log(R/R_ref_) manner as they approach targets (Rasmussen et al., 2002; Au and Benoit-Bird, 2003; Au and Würsig, 2004; Madsen and Surlykke, 2013). However, this still leaves a 20 log(R/R_ref_) increase in EL with reduced target range. Bats have been shown to handle such resulting increases in EL with decreasing range with a stapedial reflex, which is induced at every pulse emission (Kick and Simmons, 1984). This mechanism forms an auditory time-varying gain control that, in concert with the reduction in SL with reduced target range, stabilizes perceived echo intensities (Suga et al., 1974; Suga and Jen, 1975; Kick and Simmons, 1984). A similar auditory time varying gain control has been shown for toothed whales both for their own echolocation clicks and echoes, and in experiments with artificial double pulses simulating clicks and echoes (Supin et al., 2005, 2010; Li et al., 2011; Linnenschmidt et al., 2012; Finneran et al., 2013). Thus, auditory brainstem response (ABR) studies conducted by subjecting toothed whales to two sound pulses with varying delays have revealed a significant reduction in hearing sensitivity for the second pulse, called the test pulse, following the first pulse, the conditioning pulse (Popov and Supin, 1990; Supin and Popov, 1995; Popov et al., 2001; Supin et al., 2007; Supin and Popov 2015).

These findings have collectively led to the hypothesis that the observed time varying auditory gain control in toothed whales is at least in part the result of gradual release from forward masking induced by the outgoing click (Supin et al., 2008; Supin and Nachtigall, 2013). However, to what extent middle ear mechanisms such as the stapedial reflex in the case of bats, contribute to the development in hearing sensitivity following a loud emitted broadband click remains an open question (Nachtigall and Supin, 2015). Since forward masking is only acting on the auditory neurons rendered refractory by the previous stimulus, it is a highly frequency-specific phenomenon related to the width of the auditory filters and the spectra of the sound stimuli used (Popov et al. 2001). A middle ear reflex, however, should affect the overall auditory sensitivity, including frequencies not involved in eliciting the reflex, as was seen by Nachtigall and Supin (2015).

We therefore predict that for pulses played in close succession, the first (conditioning) pulse will only result in a decrease in the ABR elicited by the second (test) pulse of another frequency if there is a stapedial reflex involved. Here, we test the hypothesis of a stapedial reflex on a trained porpoise using artificial double pulses, and we show that the ABR resulting from the test pulse is masked only if it has the same frequency as the conditioning pulse. This suggests that the toothed whale auditory time varying gain control is not mediated by the middle ear, but rather by frequency dependent mechanisms such as forward masking and efferent feedback to the outer hair cells.

## RESULTS AND DISCUSSION

**Fig. 1.**
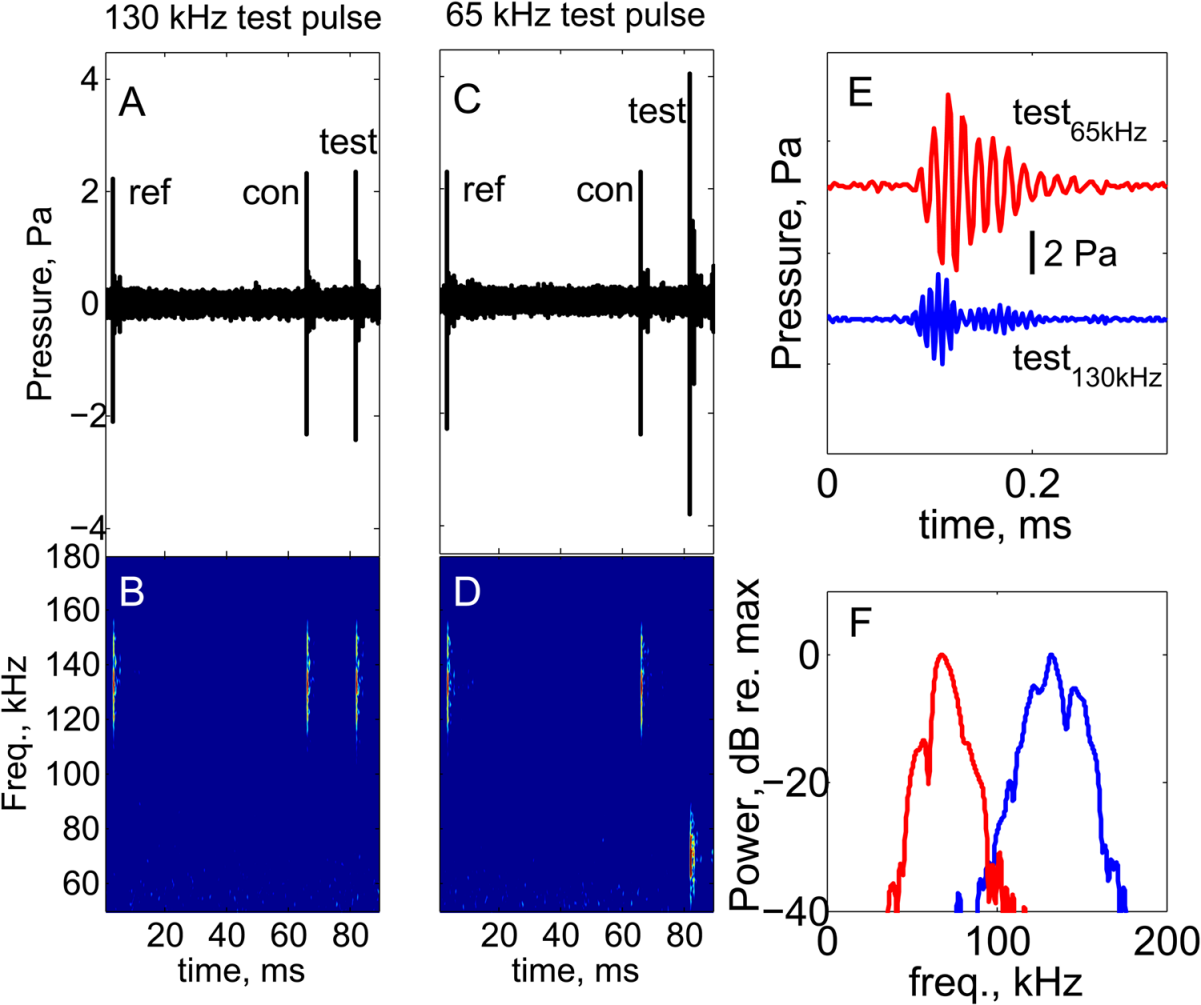
**Waveforms and spectrogram of the acoustic stimuli.** (A,B) The 130 kHz reference (ref) pulse followed by 130 kHz conditioning (con) and test (test) pulses. (C,D) The 130 kHz reference (ref) pulse followed by 130 kHz conditioning (con) and 65 kHz test (test) pulses. Note, that the time remaining before the next presentation round occurs has been omitted. The next ref pulse will occur at 126 ms from the onset of the ref pulse shown here. (E,F) Detailed test waveforms at 130 and 65 kHz (E), along with their power spectra (F).

As a result of the high degree of functional convergence in the sonar used by bats and toothed whales (Madsen and Surlykke, 2013), we hypothesized that the observed auditory time varying gain control in toothed whales could be explained in part by a stapedial reflex as has been shown to exist in bats (Suga and Jen, 1975; Kick and Simmons, 1984). Here, we tested that hypothesis by subjecting a harbour porpoise to a series of double pulses varying in inter-stimulus interval and frequency (Fig. 1). A middle ear reflex would be predicted to affect the ABR magnitude both for the 65 and the 130 kHz test pulses (Nachtigall and Supin, 2015), whereas a difference in the evoked potentials for the two frequencies would reject the hypothesis that a middle ear mechanism is responsible for the observed time varying auditory gain control in echolocating toothed whales.

The use of artificial clicks rather than the natural echolocation clicks emitted by the animal itself was necessary in this experiment, since it is unknown what is actually heard by the animal during echolocation. The right phonic lips of porpoises produce a broad band pulse that is only turned into the characteristic narrow band high frequency click after filtering through the melon (Madsen et al., 2010). Thus, echolocating porpoises likely hear a broad band pulse at a sensation level that is hard to infer, which led us to use externally generated double clicks for testing this hypothesis.

**Fig. 2.**
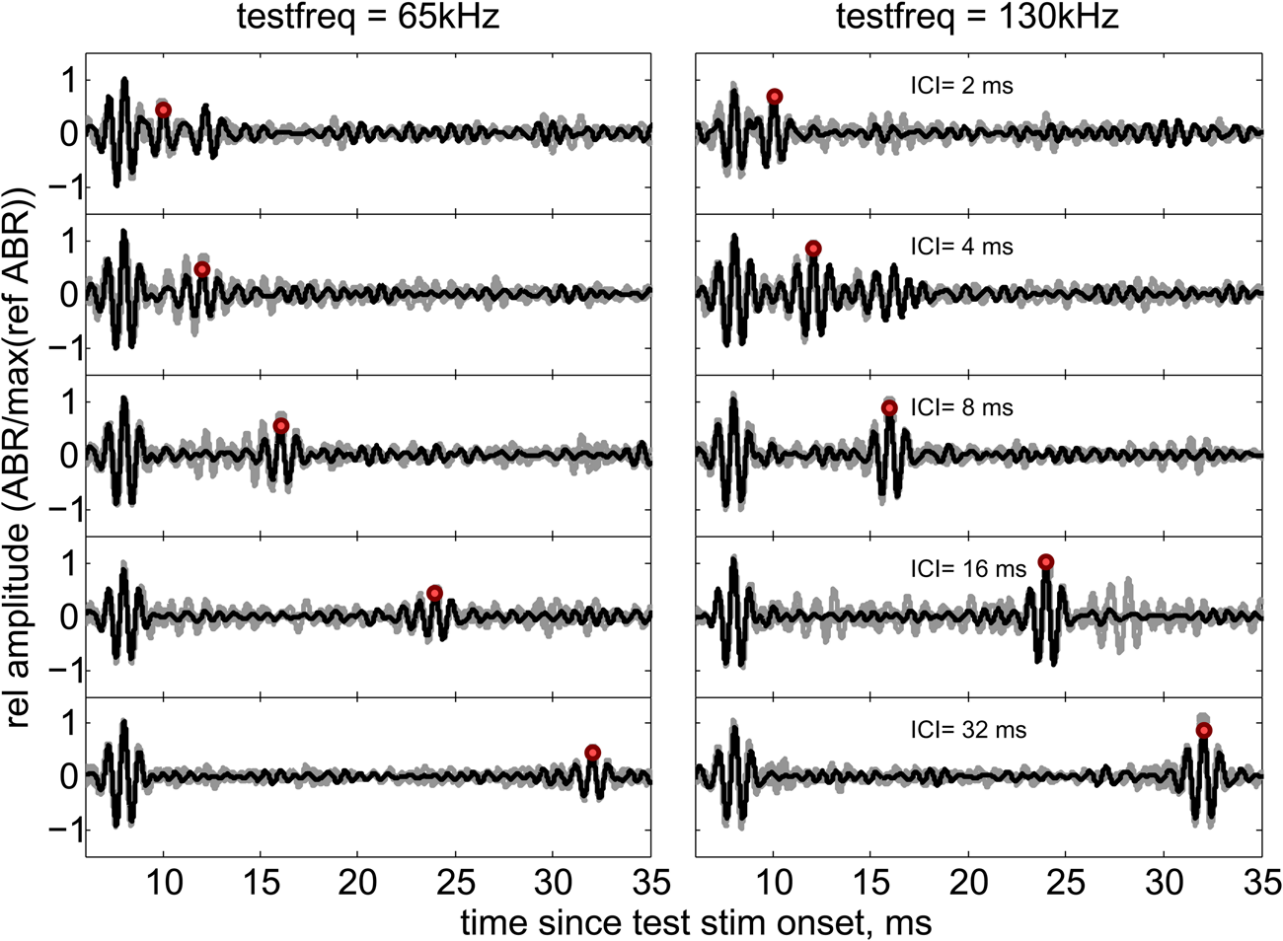
**Averaged cross-correlated ABR traces.** Mean ABR traces from each scenario within one session where the first ABR trace is evoked by the conditioning pulse at 130 kHz and the trailing ABR traces with increasing delay are evoked by either 130 kHz or 65 kHz test pulses. Red dots mark the mean response amplitude values from this session that were used for further analysis. The responses to the preceding reference pulses are not displayed in this figure.

**Fig. 3.**
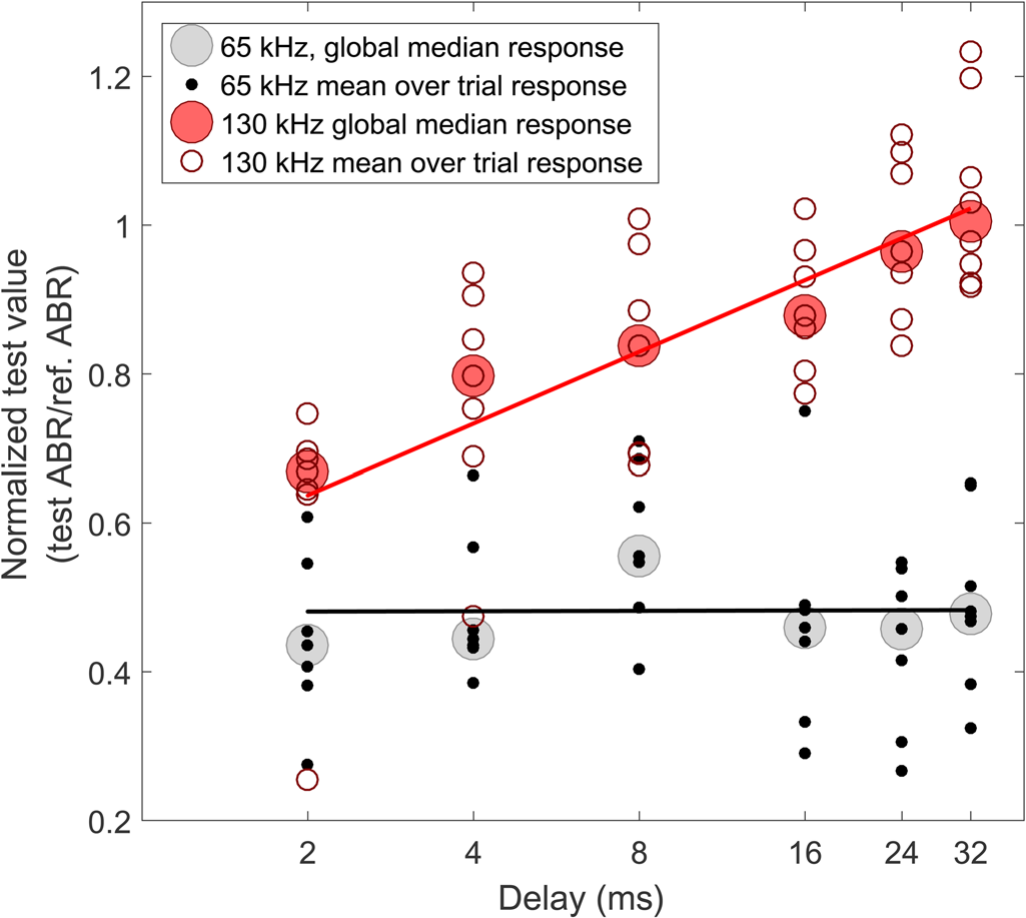
**Peak normalized cross-correlated ABR amplitude as function of the delay between conditioning and test pulses.** Response amplitude to the normalized ABR elicited by the 65 kHz test pulse is shown with black dots. Response amplitudes to the 130 kHz test pulse is shown with red circles. Response amplitude medians over all trial for each delay are indicated with shaded circles. The straight lines represent linear regressions to the trial-specific response data.

After 3 months of training the animal to station on the bite plate and accept the electrodes, the data were collected during 7 sessions on 4 days. Each session consisted of 3-4 trials of 35 s in duration. This gave 80 successive cycles for each frequency and delay combination within a single session. The shape and duration of the cross-correlated ABR response used for analysis (Fig. 2) is the result of filtering and subsequent cross-correlation of the mean signal with the similarly filtered template. The response to the 65 kHz test stimulus was consistently around half of the amplitude of the response to the 130 kHz reference signal. This effect is depicted in Fig. 3 showing the normalized responses to the test pulse as a function of delay between the conditioning and test pulses. The projected signal levels were chosen so as to provide the same sensation level according to published audiograms (Kastelein et al., 2002). Therefore, we propose that the different ABR magnitudes reflect that more neurons are dedicated the part of the basilar membrane that covers the echolocation band of porpoises between 100 and 150 kHz.

When subjecting the porpoise to conditioning and test pulses of the same frequency, the normalized test response showed a reduction in amplitude with decreasing delay (Fig. 3). At a delay of 2 ms, the normalized test response was around 0.6 of the reference response. The normalized test response steadily increased with delay until reaching a value of 1 between 8 and 24 ms (Figs 2–3). A Monte Carlo permutation test on Spearman correlation values between the delay and normalized ABR amplitude, with 10,000 iterations, showed *P*<0.005 and a correlation coefficient of 0.87, demonstrating a significant increase in test response with increasing delay.

When subjecting the porpoise to a conditioning pulse and a test pulse of different frequencies, the 130 kHz conditioning pulse did not induce any delay specific reduction in the 65 kHz test response (Fig. 3). A permutation test with 10,000 iterations for the significance of the Spearman correlation between delay and peak response amplitude was rejected by a *P*=0.6. The correlation coefficient in this case was −0.07 demonstrating no significant effect on ABR amplitude with delay. It might appear from the data shown in Fig. 3 that there is a log-linear increase in the responses to 65 kHz test stimuli with delays increasing from 2 to 8 ms. However, this increase is not significant for the 65 kHz data (*F*-test, *P*<0.5). For the responses to the 130 kHz test stimulus such a relationship does seem to exist with *P*<0.012.

Thus, we find that the evoked potential of the 130 kHz test pulse increased with increasing delay to the conditioning pulse, as also shown for a false killer whale (Supin et al., 2007) and bottlenose dolphins (Supin and Popov, 1995). The gradual recovery of the evoked potential converges on the normalized response value (i.e. 1) between 8 and 24 ms delay (Fig. 3), which is considerably longer than what has been reported for a false killer whale (3 ms) (Supin et al., 2007) and for a bottlenose dolphin (5 ms) (Popov and Supin, 1990; Supin and Popov, 1995), under similar conditions. Interestingly, a gradual reduction in hearing threshold in a bottlenose dolphin for delays corresponding to a target range of up to 80 m (some 100 ms), has been found (Finneran et al., 2013). When keeping in mind that prior masking studies, including this one, have shown a complete recovery of auditory capabilities following a stimulus in a few tens of milliseconds (Supin et al., 2007), forward masking acting on delays up to 100 ms seems unlikely. Such long recovery times may suggest narrow and hence ringing auditory filters in what has been proposed to be an acoustic fovea of porpoises (Popov et al., 2006). However, such narrow filters would not match the short and relatively broad clicks of porpoises very well and could compromise echo timing and hence effective ranging of targets. Interestingly, Kastelein et al. (2009) found an increase in critical ratio of 3.3 dB/octave, more consistent with the prediction from a constant Q filter bank. Whether porpoises have narrow or broad band filters in their auditory systems when echolocating still remains unresolved. Conversely, the response to the 65 kHz test signal showed no change in amplitude with delay (Fig. 3). This demonstrates that the 130 kHz conditioning pulse did not affect auditory sensitivity at frequencies outside its own critical band. A somewhat similar result has been found for a bottlenose dolphin, using much longer conditioning pulses, that did not resemble the animals' own echolocation clicks, at varying frequencies from 45 to 90 kHz, and a test pulse at 64 kHz (Popov et al., 2001).

Under the assumption that double pulse stimulations successfully mimic hearing during active echolocation (*sensu* Supin et al., 2007), we therefore conclude that there is no support for a hypothesis that the auditory time varying gain control is formed by a stapedial or any other middle ear reflex in echolocating toothed whales. Rather, the delay specific change in the magnitude of auditory evoked potentials elicited in double pulse studies seems better explained by forward masking due to the refractory period of the affected auditory neurons in concert with more central neural mechanisms perhaps affecting the outer hair cells (Nachtigall and Supin, 2015). Echolocating toothed whales may, contrary to bats, not be able to contract their larger stapedial muscles to move their much heavier middle ear ossicles at the required speeds to form a functional time varying control, and they can, due their sound production mechanism, possibly not have the same motor control of pulse emission timing as do bats to allow for such a reflex to be functional. They may, on the other hand, not need to have as much control, as the distance between the ears and the sound production, and the bone and air isolation of the ears is much more favorable than in small bats. It remains to be understood how toothed whales may actively employ higher order control of their hearing sensitivity in certain echo delay windows of interest.

## MATERIALS AND METHODS

The experiments were conducted at the Fjord and Bælt Centre in Kerteminde on a female porpoise, Freja, 20 years old, 160 cm long and 65 kg. The animal was trained to station on a biteplate at 1 m depth. The animal was subjected to a series of double sound pulses. Each pulse was produced by excitation of a resonant piezo transducer with a signal with an RMS duration of 50 µs, with varying delays of 2, 4, 8, 16, 24 and 32 ms between a conditioning pulse at 130 kHz, and a test pulse at either 65 or 130 kHz (Fig. 1). Despite the input durations of the electrical signals being identical, the resulting Q value (freq. centroid/centralized rms bandwidth) of both pulses as recorded in the set-up was constant at around 10. For each conditioning and test pulse pair, a reference pulse without subsequent test pulse was also transmitted (Supin and Popov, 1995). Just above the biteplate, a hydrophone (Reson TC 4034, sens: −218 dB re. 1 V/µPa) recorded the sound pulses played to the animal during the experiment. Two hydrophones 1 m in front of the stationed animal acted as the transmitters. One HS150 (Sonar Products, Beverley, East Yorkshire, UK, transmitting sensitivity 130 dB re. 1 V/µPa) was used for the 130 kHz pulse, and a HS70 (Sonar Products, transmitting sensitivity 135 dB re. 1 V/µPa) was used for the 65 kHz pulse. The two transducers were aligned vertically and placed a few centimeters apart, so only minute angular differences (<2 degrees) in the direction of each sound pulse resulted. The 130 kHz pulses were adjusted to render received levels at 78 dB re. 1 µPa^2^s [130 dB re. 1 µPa (pp)] and the 65 kHz pulses at 86 dB re. 1 µPa^2^s [135 dB re. 1 µPa (pp)]. The 8 dB higher energy for the 65 kHz pulse was chosen to compensate for the ∼8 dB lower hearing sensitivity at 65 kHz compared with 130 kHz (Kastelein et al., 2002).

All sampling and signal generation was carried out using a single multifunction USB-device (USB-6356, National Instruments, Houston, TX) running synchronously at 500 kHz for both transmission and reception of signals, controlled by a custom-made LabVIEW (National Instruments) program. Therefore, the initiation of playback and recording used the same initial trigger event, and both sampling and playback used the same controlling clock and sampling rate. Two D/A channels were set to deliver a maximum of 1 V peak, and were amplified by 20 dB using a custom-built battery-driven power amplifier to avoid overloading of the D/As when driving the hydrophones. Each output channel was monitored on a separate AD channel. The outputs from the receiving TC4034 hydrophone monitoring the signals generated was amplified 70 dB by a custom built amplifier before digitization.

ABRs were recorded during each trial using silver electrodes contained within two suction cups. Following established procedures (Popov and Supin, 1990), one suction cup was placed just posterior to the blowhole, and one was placed further back on the side of the animal. A common ground reference electrode was placed just above one of the electrodes forming contact with the water. The electrodes were connected to a differential Grass P55 amplifier with band pass filter (2 pole, 30-10,000 Hz), and with amplification set to 80 dB. The amplified output was also digitized at 500 kHz on the multifunction device. Each session would on average consist of four trials. Within each trial the animal would be subjected to 2-3 different delay and frequency combinations, so all the different combinations, except the longest delays, were contained within a single session. This was to ensure as constant an electrode placement as possible across the different trials.

All data were analyzed using custom scripts in MATLAB 2013 (MathWorks). The ABR data were band-pass filtered using an FFT-based band-pass filter from 700 to 1700 Hz, with one narrow band of electrical noise around 1 kHz filtered out using a narrow band-reject filter. The signal was split into segments that were equal in length to the inter-stimulus interval of 126 ms between each reference pulse. This interval was chosen to enable us to average out as much 50 Hz interference as possible. The resulting data matrix then contained the overlaid recorded responses to one presentation of a reference ‘ref’, conditioning ‘con’ and ‘test’ pulse. The ABR response value for each stimulus type was found by averaging 80 stimulations obtained from each frequency and delay combination. This comparatively low number of averages was necessitated by the limited time a porpoise can work in these experiments compared with larger odontocetes coupled with the need to cover all ICI values and both frequencies within a single session. The cross-correlation was calculated between the averaged result and a nearly noise-free template, formed from an average of all reference responses collected within the same session. The data series for each condition were then normalized to the peak of the cross-correlation result with the reference response peak of that particular scenario (Fig. 2). The normalized test-click responses were evaluated as the peak value in a 0.2 ms time window, centered on the relevant delay from the peak of the response to the conditioning signal. The peaks in this case do not necessarily correspond to specific brain stem structures, as it was the peak of the cross-correlation functions that was evaluated.
